# Research Progress of Phospholipid Vesicles in Biological Field

**DOI:** 10.3390/biom14121628

**Published:** 2024-12-19

**Authors:** Na Zhang, Jie Song, Yuchun Han

**Affiliations:** 1 School of Pharmacy, Binzhou Medical University, Yantai 264003, China; sisongjie00@163.com; 2 CAS Key Laboratory of Colloid, Interface and Chemical Thermodynamics, CAS Research/Education Center for Excellence in Molecular Sciences, Beijing National Laboratory for Molecular Sciences (BNLMS), Institute of Chemistry, Chinese Academy of Sciences, Beijing 100190, China

**Keywords:** phospholipid vesicles, drug delivery, cell mimics, gene therapy, biological detection, vaccine development

## Abstract

Due to their high biocompatibility, biodegradability, and facile surface functionalization, phospholipid vesicles as carriers have garnered significant attention in the realm of disease diagnosis and treatment. On the one hand, phospholipid vesicles can function as probes for the detection of various diseases by encapsulating nanoparticles, thereby enabling the precise localization of pathological changes and the monitoring of disease progression. On the other hand, phospholipid vesicles possess the capability to selectively target and deliver therapeutic agents, including drug molecules, genes and immune modulators, to affected sites, thereby enhancing the sustained release of these agents and improving therapeutic efficacy. Recent advancements in nanotechnology have led to an increased focus on the application of phospholipid vesicles in drug delivery, biological detection, gene therapy, and cell mimics. This review aims to provide a concise overview of the structure, characteristics, and preparation techniques of phospholipid vesicles of varying sizes. Furthermore, we will summarize the latest research developments regarding their use as nanomedicines and gene carriers in disease treatment. Additionally, we will elucidate the potential of phospholipid vesicles in facilitating the internalization, controlled release, and targeted delivery of therapeutic substrates. Through this review, we aspire to enhance the understanding of the evolution of phospholipid vesicles within the biological field, outline prospective research, and address the forthcoming challenges associated with phospholipid vesicles in disease diagnosis and treatment.

## 1. Introduction

Nanomaterials exhibit significant promise in the realm of disease diagnosis and treatment, attributable to their tunable dimensions and exceptionally high specific surface area [1,2]. Nanomaterials that are conjugated with pharmaceutical agents or genetic molecules can enhance the stability, solubility, and bioavailability of these substrates [3]. This is particularly advantageous in addressing the intrinsic limitations associated with therapeutic drugs, which often include inadequate water solubility, non-specific distribution within biological systems, unintended effects, and cytotoxicity towards a majority of normal cells.

Among the various nanocarriers, phospholipid vesicle carriers represent a significant category. Since the 1960s, phospholipid vesicles have served as model biological membranes for investigating the interactions between biological membranes and proteins, peptides, bacteria, drug molecules, and surfactants [4,5,6,7,8,9]. Research on the interactions between phospholipid vesicles and proteins is helpful for the purification and remodeling of membrane proteins, modification of phospholipid membranes, and drug trafficking [6,10,11,12,13,14]. To enhance the observation of phospholipid vesicle functionality within biological systems, micron-scale giant phospholipid vesicles (GUVs) have been developed [15]. In contrast to vesicles with sizes of several hundred nanometers, GUVs typically range from 1 to 100 microns in diameter and have been extensively utilized as simplified artificial cell models [16]. GUV models promote the investigation of the distribution kinetics and heterogeneity of membrane phospholipids, functionality of membrane proteins, and various biological/chemical reactions pertinent to metabolic processes in living cells, thus contributing to a deeper understanding of cellular mechanisms [8,17]. The real cell membrane also exhibits considerable complexity and diversity, and the variations in phospholipid types, cholesterol, membrane proteins, and carbohydrates will significantly influence the functionality of simulated biological membranes in vitro [18,19]. Consequently, the development of multi-component membrane systems that closely resemble real cell membranes is crucial for the in vitro examination of the interactions between cell membranes and other substances, alongside cellular processes and functions.

Cell membranes serve as a protective barrier for cells against the intrusion of foreign substances, including peptides, proteins, oligonucleotides, pharmaceuticals, and imaging agents [20,21,22]. To facilitate the transmembrane transport of therapeutic agents, such as drug molecules, biocompatible phospholipid vesicles are employed as carriers. These vesicles can encapsulate drugs, nucleic acids, and other therapeutic contents, enhancing their ability to interact with target cells through a membrane fusion mechanism during the drug loading process. This interaction promotes the effective release of the therapeutic agents, while the phospholipids themselves can be metabolized into biological membrane components without disrupting normal physiological functions [23,24]. In the medical area, phospholipid vesicles are capable of transporting both hydrophilic and lipophilic drugs due to the amphiphilic characteristics of phospholipids. Furthermore, the modification of phospholipid vesicles with small molecules, peptides, and polymers can enhance the targeting capabilities of phospholipid vesicle drug carriers [25,26]. This modification can regulate the permeability of phospholipid membranes, improving the selective permeability of the vesicles and thus facilitating the distribution of nanodrugs within diseased tissues, optimize the pharmacokinetics of the drugs, and ultimately enhance their therapeutic efficacy [27,28,29].

This review summarizes the use of phospholipid vesicles in various biomedical applications, including drug delivery, gene therapy, and disease detection. The first section covers the structural features and fundamental properties of phospholipids, as well as the methods for preparing phospholipid vesicles. The second section focuses on various strategies to enhance the functionality of these vesicles for biomedical purposes, such as modifying their permeability with targeted molecules for the precise release of their contents and utilizing them in gene therapy and immune regulation for responsive treatments. Based on their good biocompatibility and facile surface modifications, phospholipid vesicles, through covalent or non-covalent interactions, are essential for advancing the applications of nanocarriers in disease treatment.

## 2. Phospholipid Vesicles

### 2.1. Structural Characteristics of Phospholipids

**Figure 1:**
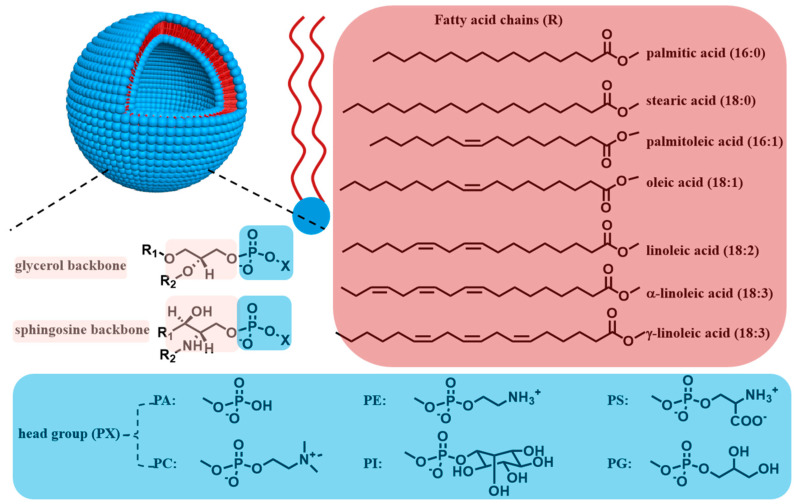
Schematic representation of the classification of phospholipids, which are used to prepare phospholipid vesicles. PA, phosphatidic acid; PC, phosphatidylcholine; PE, phosphatidylethanolamine; PS, phosphatidylserine; PG, phosphatidylglycerol; PI, phosphoinositide.

Phospholipids represent the predominant and critical constituents of cellular membranes [30,31]. Their amphiphilic molecular architecture facilitates the formation of a bilayer structure, which effectively impedes the intrusion of external substances. Phospholipids are synthesized through the covalent bonding of various hydrophilic head groups to one or two hydrophobic fatty acid chains, mediated by phosphate group clusters [32] (Figure 1). These molecules can be categorized into two primary classes: glycerophospholipids and sphingomyelins [33]. Sphingomyelin is predominantly located in neural and cerebral tissues [34,35]. It is composed of sphingosine, fatty acids, and phospholipid choline. Sphingosine is characterized as an amino unsaturated diol with an extended carbon chain in its aliphatic group. The hydroxyl group at carbon 1 is connected to phosphocholine via a phosphate bond, while the amino group at carbon 2 is linked to fatty acids through an amide bond [36]. The predominant form of sphingosine in the human body contains 18 carbon atoms [37]. However, the fatty acid constituents of sphingomyelin exhibit variability across different tissues and organs [38]. For instance, stearic acid, tetracosenic acid, and nervonic acid are primarily found in neural tissues, whereas palmitic acid and eicosapentaenoic acid are prevalent in spleen and lung tissues, respectively [37]. Glycerophospholipids, the most abundant phospholipids in cellular membranes [39], are composed of one glycerol molecule, one phospholipid molecule, two higher fatty acid molecules, and one organic molecule. Based on the chemical structure of their polar head groups, glycerophospholipids can be classified into several categories, including phosphatidic acid (PA), phosphatidylcholine (PC), phosphatidylethanolamine (PE), phosphatidylserine (PS), phosphatidylglycerol (PG), and phosphoinositide (PI) [33,40,41]. Furthermore, the two fatty acid chains in phospholipids may be identical or distinct and can be either saturated or unsaturated. Natural phospholipids typically contain unsaturated fatty acid chains. The fatty acid chains of glycerophospholipids generally consist of an even number of carbon atoms, typically ranging from 16 to 24 carbon atoms in length [42]. Consequently, the variation in the hydrophobic chain length, the unsaturation nature, and the head group type can yield an extensive array of phospholipid molecules with unique structural and chemical characteristics.

### 2.2. Basic Properties of Phospholipids

Despite the fact that phospholipid structures are not identical, they all possess a fundamental property—self-assembly [43,44,45]. When dissolved in water, phospholipid molecules spontaneously assemble into vesicles with the polar and hydrophilic head groups extending into the water, while the non-polar and hydrophobic chains gather to form a thermodynamically stable bilayer [46]. The variations in phospholipid structures also influence the properties of the resulting bilayer. Different polar head groups lead to distinct charges, which are determined by the sum of phosphate’s negative charges and the positive charges of the polar substituents when dispersed in a water solution at pH 7.0. For instance, when choline or ethanolamine are present as polar substituents, their positive charges are neutralized by phosphate’s negative charge, resulting in zero net charge for PC and PE phospholipids. On the other hand, serine simultaneously carries positive and negative charges, causing PS phospholipids to carry one unit of negative charge [41,47]. Similarly, glycerol, as a non-charged polar substituent, causes PG-type phospholipids to carry one unit of negative charge due to phosphate’s negative charge. Consequently, PS and PG-type phospholipids are commonly referred to as anionic phospholipids [48,49,50,51].

The characteristics of phospholipids, such as the head group, the length of the hydrophobic chain, and the degree of unsaturation, significantly influence the molecular arrangement and fluidity of phospholipid membranes. A key physical property in the assembly of phospholipids is the phase transition temperature (*T*c), which is the temperature at which the fatty acid chains shift from a liquid crystal state to a gel state [52]. At this temperature, the activity of the phospholipid alkyl chains increases, leading to greater membrane permeability. Generally, longer hydrophobic alkyl chains result in a higher *T*c [53], while a higher unsaturation degree lowers the *Tc* [54]. The type of headgroup also has a minor impact on the *T*c [55]. For instance, phospholipids with PS and PG head groups exhibit similar *T*c values (Table 1), both of which are higher than those of phospholipids with a PC head group. Additionally, the structure of the phospholipid backbone influences the *T*c, with sphingomyelin (ESM) having a relatively high *T*c [34]. The *T*c is crucial for the stability of phospholipid vesicles. When creating vesicles, it is essential to consider the storage conditions and practical applications for selecting phospholipids with suitable phase transition temperatures.

### 2.3. Preparation of Phospholipid Vesicles

**Figure 2:**
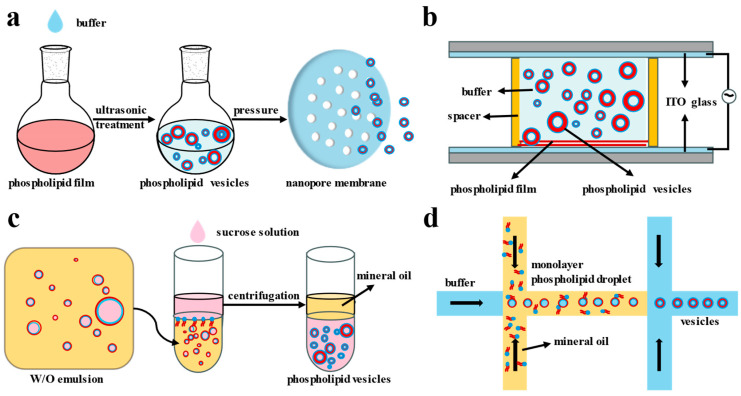
Engineering strategies for constructing phospholipid vesicles. (**a**) Extrusion method. (**b**) Electrochemical formation method. (**c**) Emulsion phase transfer method. (**d**) Microfluidic method.

The homogeneous, controllable shape and fluidity of phospholipid vesicles make them an ideal model and carrier for studying biophysical, biochemical, biomedical, and cellular processes. To better mimic the tissues, organelles, and cells of various sizes found in living systems, phospholipid vesicles can be prepared as single and multilayer structures ranging from tens of nanometers to tens of microns using various techniques. Currently, the preparation of phospholipid vesicles is primarily achieved through the methods of extrusion, emulsion phase transfer, electrochemical techniques, microfluidics (Figure 2) [17,66,67], and the Mozafari method [68]. The goal of these methods is to facilitate the self-assembly of phospholipid molecules under controlled external conditions.

#### 2.3.1. Extrusion Method

Quantitative phospholipids are weighed and dissolved in an organic solvent, typically chloroform or a chloroform-methanol mixture. The organic solvent is subsequently removed using a rotary evaporator, resulting in a uniform phospholipid film adhering to the inner wall of the flask. This phospholipid membrane is then subjected to a vacuum drying oven for a minimum of two hours to ensure the complete removal of the organic solvent. Following this, the phospholipid membrane is hydrated with a specified volume of aqueous phase and subjected to vortex or ultrasonic treatment to achieve thorough hydration. The initial product of the hydrated vesicles is then introduced into a phospholipid extruder. At temperatures exceeding the phase transition temperature of the utilized phospholipid and under applied external force, the mixture is extruded through a filter membrane with a pore size smaller than the vesicle dimensions, which is repeated ten times. This procedure facilitates the extrusion, deformation, rupture, and self-assembly of the phospholipids, yielding monolamellar vesicles that are smaller and more uniform in size. The aqueous solution containing the phospholipid vesicles can be subjected to repeated extrusion to achieve vesicles of the desired size [69] (Figure 2a). For the preparation of smaller phospholipid vesicles, the sequential extrusion through membranes with progressively smaller pore sizes is recommended [9]. The extrusion method can produce monolamellar vesicles ranging from 50 to 1000 nm in size, characterized by their small dimensions and avoidance of organic solvents, which contribute to their high drug encapsulation and retention efficiencies. Furthermore, phospholipid vesicles generated through this method are frequently employed as model cell membranes for investigating the interactions between biological membranes and various molecules, including proteins, peptides, and pharmaceuticals.

#### 2.3.2. Electrochemical Formation Method

Phospholipids dissolved in an organic solvent are incrementally introduced to indium tin oxide (ITO)-coated conductive glass, resulting in the formation of a thin phospholipid film upon the evaporation of the organic solvent. The conductive glass is then secured within a rectangular pool to create a closed system, into which an aqueous phase is subsequently injected. A second piece of ITO-coated conductive glass is placed atop the first, thereby establishing a parallel plate capacitor configuration. The application of an alternating current electric field facilitates the formation of phospholipid vesicles (Figure 2b). Through electrochemical processes, a solution of GUVs ranging from 1 to 100 μm can be produced [70,71]. Initially, this technique is limited to the generation of partially amphoteric phospholipid solutions yielding giant vesicles with low ionic strength. However, advancements in electrochemical formation methodologies have emerged, enabling the production of single-layer giant vesicles and negatively charged GUVs in buffer solutions with high ionic strength [72].

#### 2.3.3. Emulsion Phase Transfer

Phospholipids are initially solubilized in a minimal quantity of organic solvent, such as chloroform or methanol, to which a stable organic solution, such as liquid paraffin or mineral oil, is subsequently incorporated. The organic solvents, specifically chloroform or methanol, are entirely evaporated through heating for at least two hours. Following this, a small volume of pre-encapsulated inner phase solution is introduced into the phospholipid oil phase, allowing for the formation of a water-in-oil emulsion through brief vortexing. The resulting phospholipid emulsion is then gradually combined with glucose and other external phase buffer solutions, facilitating the slow formation of a phospholipid bilayer at the oil–water interface (Figure 2c). After high-speed centrifugation, the upper oil phase is removed, yielding GUVs characterized by a favorable monodispersity ranging from 5 to 50 microns. These GUVs, produced via emulsion phase transfer, serve as models for artificial cells, enabling the investigation of intracellular enzymatic reactions and biosynthetic processes [73].

#### 2.3.4. Microfluidic Method

The microfluidic approach primarily relies on the emulsion phase transfer technique, utilizing a microfluidic apparatus to facilitate precise and continuous phase transfer at the oil–water interface, thereby enabling the synthesis of GUVs [74,75] (Figure 2d). By manipulating the flow rate of the solution introduced into the microfluidic device and altering the geometry of the device itself, GUVs with controllable structural characteristics and dimensions can be effectively generated. The monolayer GUVs produced through this microfluidic methodology demonstrate significant monodispersity and high encapsulation efficiency.

#### 2.3.5. Mozafari Method

The Mozafari method is an improved heating method for the preparation of phospholipid vesicles that is characterized by the absence of organic solvents during the preparation process [68]. Phospholipid vesicles produced by the Mozafari method are typically employed for the encapsulation of various substances, including drugs. In a heat-resistant container, a combination of bioactive compounds that would be encapsulated within phospholipid vesicles, along with polyols such as glycerol, propylene glycol, or sorbitol, is subjected to heating at temperatures ranging from 40 to 60 °C. Following this initial heating, phospholipids are introduced into the mixture. The mixture in the container is conducted under an inert gas atmosphere, such as nitrogen or argon, at a temperature exceeding the phase transition temperature of the phospholipids. This heating process is facilitated by stirring at a rate of 1000 rpm using a hot plate stirrer. To achieve phospholipid vesicles with a high encapsulation efficiency of bioactive compounds, the heating is maintained for a duration of one hour. It is crucial to emphasize that the phospholipid vesicles should only be stored after the temperature of the mixture has been appropriately reduced [76].

### 2.4. Characterization of Phospholipid Vesicles

Prior to the biological application of phospholipid vesicles, it is essential to thoroughly characterize their physicochemical properties, including their size, size distribution, surface charge, morphology, and physical stability [77]. Phospholipid vesicles with a size range of 50–200 nm exhibit a relatively prolonged circulation time in vivo [77], which hinders the rapid metabolism of drugs and other molecules carried inside the phospholipid vesicles from the bloodstream. Furthermore, the physical stability of phospholipid vesicles can be evaluated by monitoring alterations in their size and size distribution over time. Therefore, characterizing the size and size distribution of phospholipid vesicles is paramount before the application of phospholipid vesicles. Dynamic light scattering is the predominant technique employed to characterize the size and size distribution of phospholipid vesicles. Additionally, the morphology of phospholipid vesicles can be assessed through transmission electron microscopy and cryo-transmission electron microscopy [9]. For micron-sized GUVs, the morphology and fluidity of phospholipid vesicles in their surrounding medium are typically examined using confocal laser scanning microscopy [7]. The surface charge of phospholipid vesicles serves as an indicator of their stability in the medium. A higher surface charge enhances the electrostatic repulsion of phospholipid vesicles, thereby preventing their natural aggregation. The zeta potential of phospholipid vesicles can be quantified by electrophoretic light scattering, providing insights into their surface charge and intensity.

## 3. Application of Phospholipid Vesicles in Biological Field

**Figure 3:**
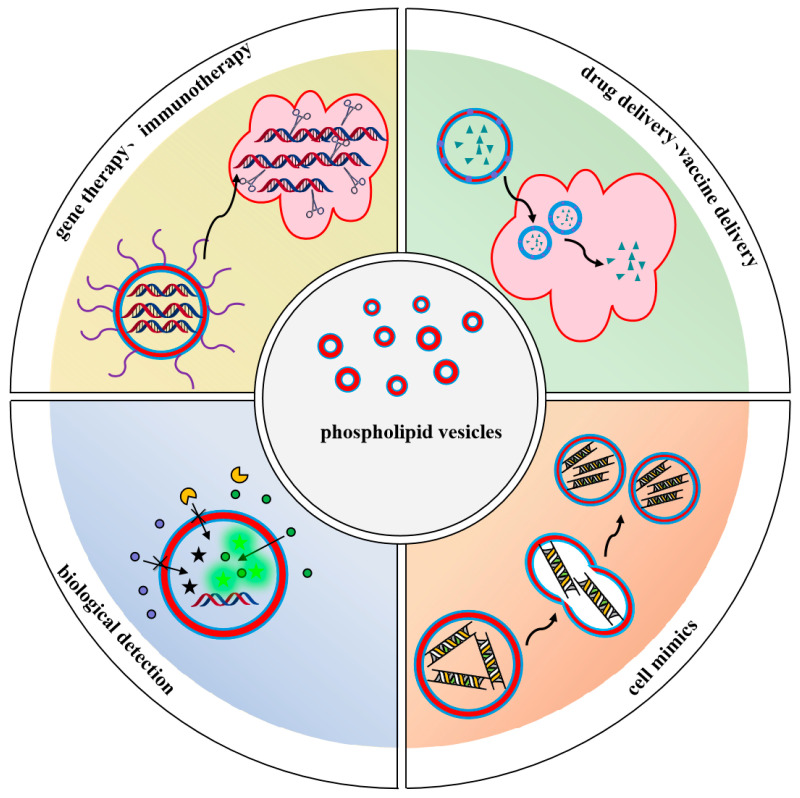
Schematic illustration of phospholipid vesicles for various biological applications.

In recent years, phospholipid vesicles have gained significant attention in the field of biomedicine due to their high biocompatibility, biodegradability, low toxicity, and ease of modification. Phospholipid vesicles, which are the predominant components of cell membranes, are frequently employed as biomimetic systems, demonstrating considerable potential in various applications [78] such as drug delivery, gene therapy, biological detection, and cell mimics (Figure 3). In the selection of phospholipid vesicles for biological applications, it is essential to choose the appropriate vesicles based on specific requirements. Neutral phospholipids, including phosphatidylcholine, sphingomyelin, and phosphatidylethanolamine, which constitute the primary components of cellular membranes, are frequently employed as models for biomembranes to investigate the interactions between cell membranes and various substances [9]. The formation of vesicles from negatively and positively charged phospholipids can enhance stability and charge through electrostatic repulsion, thereby facilitating interactions with charged molecules or cells. Small phospholipid vesicles, with diameters less than 0.1 μm, are particularly suitable for drug delivery or biosensor applications, as they can effectively target specific cells or tissues. Conversely, larger phospholipid vesicles, ranging from 0.1 to 1 μm in diameter, exhibit high encapsulation efficiency for water-soluble drugs and are appropriate for encapsulating larger molecular drugs. GUVs, exceeding 1 μm in diameter and resembling cell size, are advantageous for constructing artificial cells. These vesicles are easily observable under microscopy and can be utilized to study the mechanical properties of cell membranes, phase separation, and lipid raft dynamics. Furthermore, when GUVs are combined with other biomolecules, they can simulate intracellular metabolic reactions, gene expression-regulated protein synthesis, and the self-replication of genetic material [73,79,80]. Consequently, it is crucial to select the appropriate preparation method and phospholipid type to create vesicles that align with the specific biological application.

### 3.1. Cell Mimics

**Figure 4:**
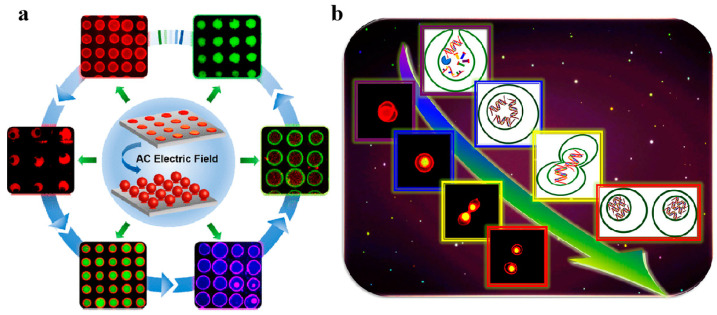
(**a**) Schematic illustration of a collection of large phospholipid vesicles generated through an electrochemical technique, designed to simulate the transmembrane transport of various species and the metabolic processes occurring within cells. Reproduced with permission from [71]. Copyright 2018, American Chemical Society. (**b**) A schematic representation illustrating the amplification of genetic information and the process of cell mitosis within deformable GUVs. Reproduced with permission from [82]. Copyright 2017, American Chemical Society.

The cell is the basic unit of life. Within living organisms, metabolic processes such as protein synthesis, genetic material self-replication, and energy transfer are localized to occur in the cell membrane. Thus, the development of complex organs, including muscle, bone, and placenta in multicellular organisms [81], as well as essential cellular functions such as protein transport, membrane trafficking, and exocytosis, necessitate the fusion of biomembranes and the interaction between proteins and biomembranes. The intricate nature of living systems presents challenges in quantifying intracellular biochemical reactions and comprehending cellular processes in vivo. To enhance the understanding of cellular mechanisms and the regulation of biochemical reactions, researchers have engineered artificial cells as simplified models to replicate specific functions and processes of authentic cells in vitro [79]. Phospholipid vesicles are frequently employed as cell-sized reactors capable of encapsulating biochemical reaction systems for the construction of in vitro cell models [73]. Han et al. [71] utilized a micro-contact exfoliation technique combined with electrochemical formation to create GUVs ranging from 20 to 100 μm in size, serving as a cell model to investigate the interaction between the membrane protein bee toxin and phospholipid membranes (Figure 4a). Their findings indicated that the transmembrane transport of substances mediated by bee toxin was dependent on the concentration of the membrane protein. Additionally, they employed a phospholipid vesicle array to simulate intracellular enzymatic reactions, incorporating horseradish peroxidase into the array to model cellular metabolic reactions. The substrate hydrogen peroxide and o-phenylenediamine were successfully diffused into the GUVs, resulting in the production of the fluorescent compound 2,3-diaminophenazine. Han et al. [82] also applied osmotic pressure to induce the deformation of the GUVs, incorporating genetic material into the cell model, thereby simulating the replication of genetic information and cell division within the nucleus of eukaryotic cells through chain polymerization reactions (Figure 4b).

**Figure 5:**
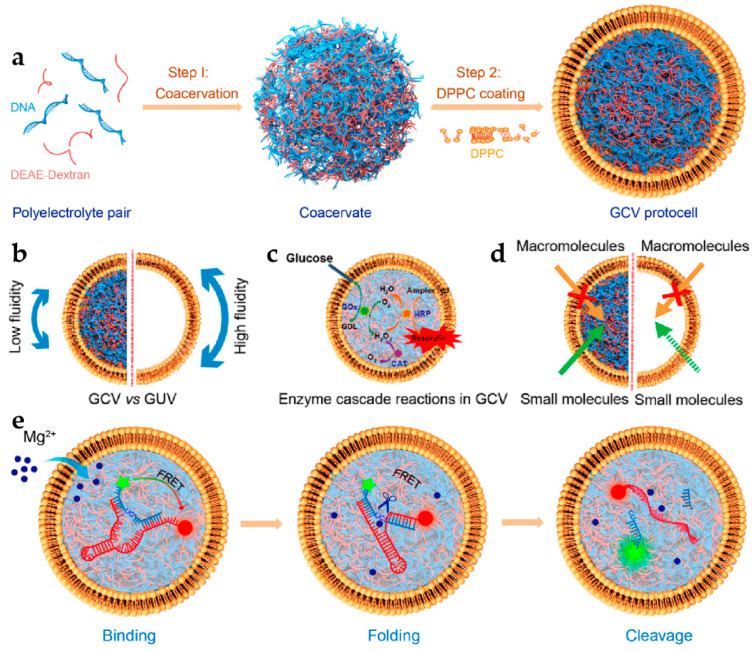
(**a**) Schematic representation illustrating the formation of giant coacervate vesicles (GCVs) through liquid−liquid phase separation. (**b**,**d**) Schematic illustration of the effect of the presence or absence of condensates in GUVs on (**b**) the membrane fluidity and (**d**) membrane permeability. (**c**) Schematic illustration of the enzyme cascade reactions induced by glucose in GCVs. (**e**) Schematic illustration of a Mg^2+^-triggered ribozyme cleavage reaction in GCVs. Reproduced with permission from [83]. Copyright 2021, American Chemical Society.

Giant monolayer phospholipid vesicles, serving as artificial cell models, also exhibit several limitations, including low membrane permeability, inadequate encapsulation rates, and structural instability. To address these deficiencies, researchers have initiated the development of artificial models that more closely resemble complex biological cells, characterized by distinct regions and compartments with varying compositions and physical properties. Liu et al. [83] employed nucleic acid/diethylaminoethyldextran to create a pre-formed coacervate, onto which dipalmitoyl lecithin was self-assembled to establish a phospholipid bilayer membrane, resulting in the formation of giant coacervate vesicles (GCVs) (Figure 5a). In comparison to traditional monolayer GUVs, the diffusion coefficient of phospholipid molecules within the GCVs was diminished by approximately 83%. Additionally, the fluorescence polarization value of the hydrophobic layer exhibited an increase of around 111%, and the electrostatic interactions between the positively charged polymer condensed phase and the negatively charged phosphate groups of the amphoteric phospholipids enhanced the binding of the external phospholipid bilayer to the inner condensed phase, thereby decreasing membrane fluidity (Figure 5b). The structural support provided by the internal aggregates enabled the phospholipid vesicles to maintain stability under high ionic strength conditions. Furthermore, small molecules with varying charges within the GCVs were able to permeate the phospholipid bilayer into the condensed phase [83]. The influx rate of these small molecules diminished as the phospholipid membrane content increased relative to non-membrane condensates. This phenomenon creates a closed chamber environment for macromolecules, effectively preventing their entry into the condensed phase. The substances with molecular weights below 4 kDa were able to traverse the phospholipid membrane into the condensed phase [83], indicating that the phospholipid membrane is anchored to the surface of the condensed phase, which enhances the membrane’s permeability and exhibits size-selective characteristics (Figure 5d). The liquid–liquid phase separation within the coagulated group of the artificial cell model provides a dense chamber environment conducive to biochemical reactions. In contrast to conventional GUVs, glucose can facilitate the glucose-horseradish peroxidase cascade within the condensed phase through the phospholipid membranes of highly selective permeability GCVs (Figure 5c). The production of resorcinol from this cascade reaction increases with enhanced glucose permeability, while the depletion of hydrogen peroxide by catalase, necessary for this cascade, will halt the glucose-mediated production of resorcinol within the artificial cell [83]. Resorcinol is primarily generated within the agglomerate and is uniformly distributed throughout the co-aggregate, exhibiting minimal permeability through the phospholipid membrane. As an artificial cell microreactor, the magnesium-mediated cleavage of hammerhead ribozymes can also be accomplished within a phospholipid vesicle-encapsulated condensed phase (Figure 5e). Compared to a free buffer solution, the catalytic activity of ribozymes exhibited an enhancement of approximately 42% in the GCVs with magnesium ion penetration, and thus, the efficiency of ribozyme cleavage was observed to increase by approximately 114%.

**Figure 6:**
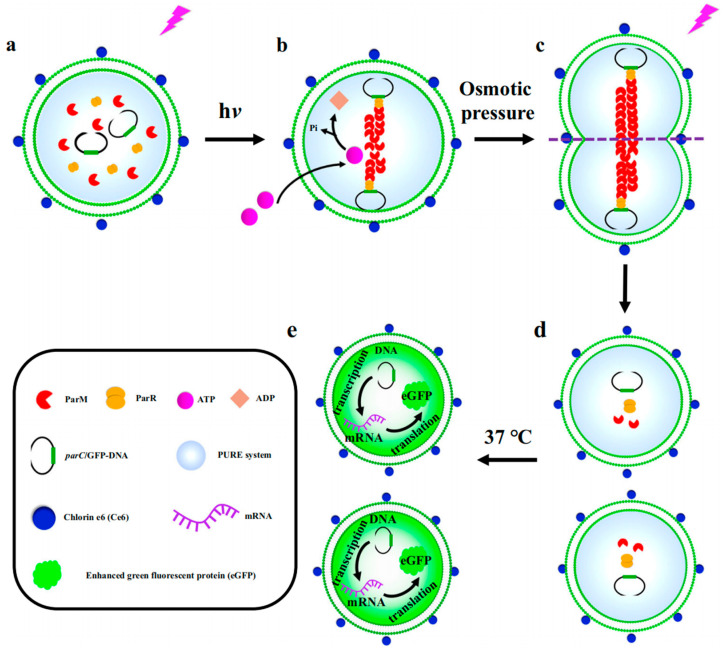
Schematic illustration of (**a**) a GUV containing elements for bacterial DNA segregation, (**b**) DNA segregation induced by ATP under the influence of laser irradiation and chlorin e6, (**c**) a deformed GUV under osmotic pressure, (**d**) the separated two daughter vesicles containing DNA similar to the original GUV, and (**e**) expressed eGFP inside the two daughter vesicles. Reproduced with permission from [80]. Copyright 2024, Jingjing Zhao, Xiaojun Han.

In addition to facilitating enzymatic reactions, the expression and transmission of genetic material, the transport of substances, and various metabolic processes in eukaryotic organisms, phospholipid vesicles can also serve as models for simulating biological processes in prokaryotes, including bacterial infection and invasion. To elucidate the antimicrobial mechanisms of peptide surfactants, we investigated the interactions between phospholipid vesicles and gemini peptide surfactants with distinct aggregation structures utilizing isothermal titration microcalorimetry [9]. Furthermore, we synthesized GUVs decorating gemini surfactants through the emulsion phase transfer method to model cellular interactions with *Escherichia coli* [7]. The adhesion and endocytosis of bacteria on the surface of these GUVs were effectively visualized using laser confocal microscopy. Our findings provide valuable insights for the rational design of antimicrobial agents and enhance the understanding of pathogen invasion mechanisms. Han et al. [80] employed the emulsion phase transfer method to create GUVs that encapsulate a bacterial DNA isolation system (Figure 6a), thereby mimicking the isolation and inheritance of bacterial plasmids. Utilizing laser irradiation and chlorin e6, the permeation of adenosine triphosphate (ATP) facilitated the polymerization of actin-like proteins across phospholipid membranes, leading to the gradual localization of plasmids towards the poles of the artificial cell (Figure 6b). Under the combined influence of osmotic pressure and laser irradiation, the GUVs underwent deformation and ultimately divided into two daughter vesicles, with the genetic material evenly distributed between them (Figure 6c,d). At physiological temperatures, the daughter vesicles inherited the genetic material from the mother GUVs, and the GUVs containing the recombinant element system were capable of transcribing and translating the enhanced green fluorescent protein (eGFP) gene, resulting in the expression of eGFP in the dividing daughter vesicles (Figure 6e).

### 3.2. Gene Therapy and Immunotherapy

**Figure 7:**
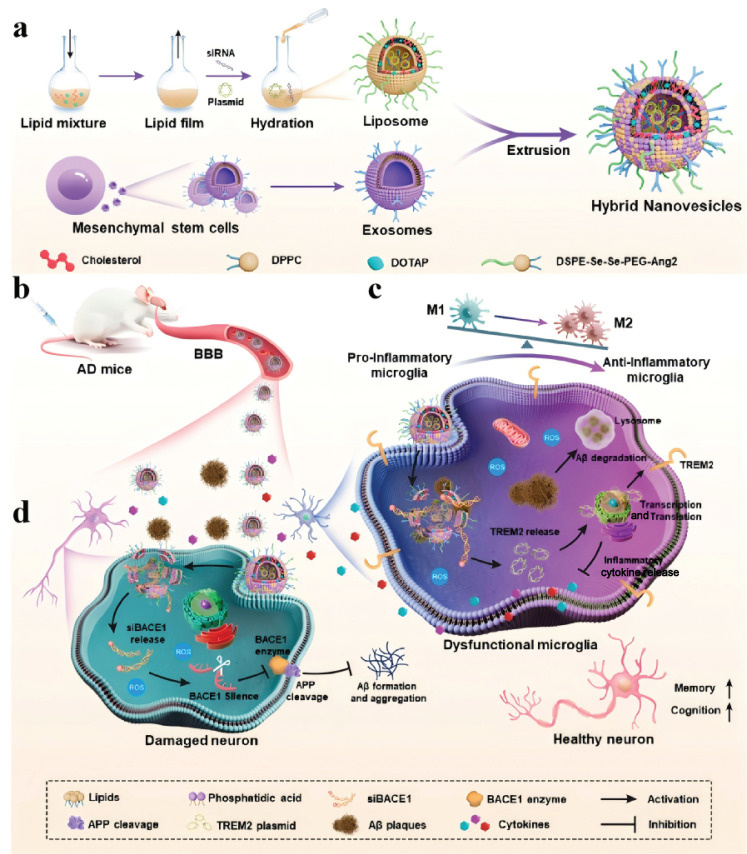
(**a**) Schematic representation of the preparation of exosome–phospholipid heterozygous vesicles that encapsulate dual genetic material. (**b**) Schematic diagram of heterozygous phospholipid vesicle carriers entering cells through the blood–brain barrier. Schematic representations of the mechanisms of (**c**) releasing the TREM2 gene in microglial cells to degrade Aβ and (**d**) delivering the BACE1 gene into neuronal cells to reduce the production of Aβ by the hybrid phospholipid vesicle transport vector. Reproduced with permission from [87]. Copyright 2024, American Chemical Society.

Gene therapy represents a therapeutic approach that involves the introduction of exogenous normal genes into target cells, aiming to modify or regulate gene expression for dealing with diseases resulting from genetic defects and abnormalities. The efficacy of gene therapy is significantly dependent on the development of safe and effective gene vector delivery systems, which remains a critical challenge in the field [84]. Presently, gene therapy vectors are categorized into viral and non-viral types. Non-viral vectors have gained considerable attention due to their superior biocompatibility, reduced immunogenicity, and enhanced safety profiles when compared to their viral counterparts. Among the various non-viral delivery systems, nanophospholipid vesicles have emerged as a promising area of research, attributed to their distinctive advantages such as surface modification capabilities, size controllability, tunable permeability, low toxicity, stability, and the capacity to transport both hydrophilic and hydrophobic substances [85,86]. In a notable study, Wang et al. [87] developed biomimetic hybrid phospholipid vesicles designed to deliver dual nucleic acids for synergistic gene therapy targeting Alzheimer’s disease. These vesicles, modified with angiopep-2 (Ang2), which encapsulated small interfering RNA targeting β-site amyloid precursor protein cleavage-1 (BACE1) and myeloid cell 2 (TREM2) plasmids, were prepared through hydration and subsequently fused with exosomes derived from mesenchymal stem cells (MSCs) to create biomimetic hybrid nanovesicles via extrusion (Figure 7a). The incorporation of exosomes and Ang2 peptides facilitates the ability of these hybrid phospholipid vesicles to traverse the blood–brain barrier, thereby enhancing drug accumulation at the sites of Alzheimer’s disease lesions (Figure 7b). Within microglial cells, the hybrid phospholipid vesicles release the TREM2 plasmid, leading to an upregulation of TREM2 expression levels, which encourages a transition of microglia from a pro-inflammatory M1 phenotype to an anti-inflammatory M2 phenotype, thereby restoring their phagocytic function towards amyloid beta (Aβ) (Figure 7c). In neuronal cells, the hybrid phospholipid vesicles deliver BACE1 small interfering RNA, resulting in the downregulation of the β-site amyloid precursor protein cleavage enzyme-1 gene, which impedes the processing of Aβ precursor protein and diminishes the production of Aβ plaques at their source, thereby further augmenting the efficacy of the synergistic treatment for Alzheimer’s disease (Figure 7d).

**Figure 8:**
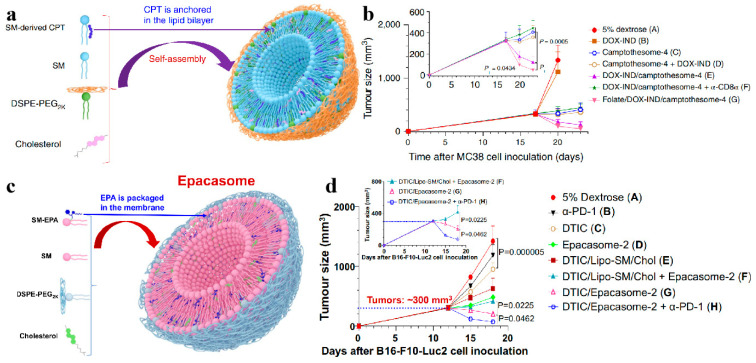
(**a**) Schematic illustration of the formation of a camptothesome. (**b**) Comparison of anti-tumor efficiency under different drug treatments. Reproduced with permission from [89]. Copyright 2021, Zhiren Wang et al. Under exclusive license to Springer Nature limited. (**c**) Schematic illustration of the formation of an epacasome. (**d**) Average tumor growth size after different drug administrations. Reproduced with permission from [90]. Copyright 2023, Zhiren Wang et al.

Immunotherapy aims to achieve anti-tumor effects by regulating the body’s immune system. Immune checkpoint blockade therapy occupies an important position in the field of immunotherapy and has great potential for cancer treatment [88]. Lu et al. [89] covalently linked neurosphingomyelin to camptothecin (CPT) through small molecule linkers and synthesized four sphingomyelin-derived CPT molecules with different structures to construct CPT nanovesicles (Figure 8a). Compared with free CPT, sphingomyelin-derived CPT nanovesicles increased the maximum tolerated dose of CPT by 5- to 23-fold in healthy mice without causing systemic toxicity. The CPT nanovesicles have an extended circulating half-life in the body, allowing them to penetrate deep into the tumor and quickly release approximately 23-fold more drugs at the tumor site. It was found that the level of immune checkpoints within tumors was upregulated after the action of CPT nanovesicles. The covalent linkage of the indoleamine 2,3-dioxygenase (IDO1) inhibitor indoximod (IND) with doxorubicin (Dox) can promote the encapsulation efficiency by 4-fold of IND in CPT nanovesicles. When targeted with folic acid, the uptake efficiency of Dox-IND/CPT nanovesicles in tumors was improved, the IDO1 pathway was significantly inhibited, and the tumor suppression effect was enhanced. In contrast, co-delivery of immune checkpoint inhibitors enhanced the anti-colorectal cancer efficacy of CPT nanovesicles (Figure 8b). Lu et al. [90] used the same method to covalently link the IDO1 inhibitor epacadostat (EPA) to sphingomyelin through a hydrazone-ester bond to construct phospholipid vesicles (Figure 8c). Compared with phospholipid vesicles that physically encapsulate EPA, sphingomyelin-derived EPA nanovesicles have significantly improved stability and drug loading capacity by approximately 16-fold. Compared with free EPA, EPA nanovesicles maintain higher stability in the circulatory system, accumulate and release at tumor sites through clathrin-mediated endocytosis, and enhance IDO1 inhibition and T cell proliferation. As a potent immunostimulant, EPA nanovesicles reverse tumor immunosuppression by enhancing IDO1 inhibition, resulting in better anti-tumor cytotoxic T lymphocyte effects. When co-encapsulated with immunogenic dacarbazine, the synergistic combination of EPA nanovesicles and programmed death protein 1 blockade can enhance the anti-melanoma immune effect and anti-tumor effect (Figure 8d) and can effectively prevent tumor recurrence.

### 3.3. Vaccine Development and Administration

With the authorization of two mRNA vaccines for immunization against the novel coronavirus, complex phospholipid vesicles have emerged as the most advanced and effective technology for mRNA delivery [86,91]. Ionizable cationic phospholipids play a crucial role in enhancing the efficiency of mRNA delivery and transfection. These phospholipids facilitate the formation of stable complexes through electrostatic interactions with negatively charged nucleic acids, such as mRNA or siRNA. Furthermore, in the acidic medium of endosomes, ionizable cationic lipids can become protonated, acquiring a positive charge that facilitates the escape of phospholipid vesicles from endosomes and the subsequent release of nucleic acids into the cytoplasm [92]. Phospholipid vesicles not only enhance the stability of mRNA, thereby protecting it from enzymatic degradation in vivo, but also facilitate the intracellular expression of mRNA. By encapsulating mRNA within phospholipid vesicles, it is possible to modify the biological distribution, cell targeting, and uptake mechanisms of mRNA, thereby promoting effective mRNA delivery and vaccine administration [93]. Among the liposomal vaccines currently available, GlaxoSmithKline Shingrix, which is designed to prevent varicella-zoster virus infection, is the most recognized. It is noteworthy that commercial vaccine products typically incorporate phospholipid components to varying degrees, and numerous liposomal vaccines are presently undergoing clinical trials [92,94].

### 3.4. Drug Delivery

Phospholipid vesicles are recognized as optimal carriers for drug delivery applications. Their bilayer structures facilitate the transport of both hydrophilic and hydrophobic pharmaceuticals to the designated sites, thereby minimizing the non-specific distribution of drugs within biological systems. Additionally, these vesicles enhance the solubility of hydrophobic drugs within tumor cells, ultimately improving the bioavailability and therapeutic efficacy of the administered drugs [95].

Notably, paclitaxel liposome represents the pioneer liposomal formulation approved by the China Food and Drug Administration, as well as the first injectable paclitaxel liposome product globally [96]. The encapsulation of paclitaxel, a compound with poor water solubility, within the phospholipid bilayer of a nanomedical carrier effectively addresses the solubility challenge and significantly enhances the therapeutic outcomes associated with paclitaxel treatment. Table 2 lists some drug products on the market based on the delivery of phospholipid vesicles. From the standpoint of the formulation, functionality, characteristics, and adverse effects of currently available commercial phospholipid pharmaceuticals, addressing the clinical translation of phospholipid formulations hinges on technical challenges, including size regulation, stability enhancement, safety performance optimization, and predictive modeling of phospholipid vesicles. Successful clinical translation necessitates the availability of consistent and reproducible products. However, the majority of phospholipid vesicles utilized in clinical trials are predominantly produced in small batches, making the large-scale preparation of phospholipid vesicles with uniform size a significant challenge. Given the intricate nature of human diseases, it is imperative to develop formulations that are both size-controllable and highly reproducible for effective clinical application. In recent years, there has been a notable increase in research focused on enhancing the performance of phospholipid vesicles. The research indicates that the surface modification of phospholipid vesicles with polypeptides, polymers, and other molecules can enhance the targeting capabilities of drug carriers, mitigate the toxic side effects of pharmaceuticals on healthy tissues, and facilitate the localization of nanomaterials within pathological sites [25,97,98]. For instance, Wang et al. [99] successfully developed a low-toxicity nanomedicine carrier utilizing phospholipids derived from natural egg yolk, which demonstrated effective delivery of the anticancer agent doxorubicin and achieved significant inhibition of breast and liver cancer in murine models. Furthermore, the targeted modification with folic acid improved the distribution of carrier-mediated drugs within tumors, thereby enhancing the tumor-targeting efficacy of yolk phospholipid vesicle vectors. In another study, Yu et al. [100] synthesized choline phosphate lipid molecules based on the phospholipid choline and established a targeted drug delivery system using specific targeting peptides. This approach resulted in increased drug accumulation at tumor sites, thereby significantly enhancing drug utilization efficiency and the overall therapeutic effect against cancer.

**Table 2:** Drug products on the market based on delivery of phospholipid vesicles. HSPC, 1,2-diacyl-sn-glycero-3-phosphocholine (SOY); DSPE, 1,2-distearoyl-sn-glycero-3-phosphorylethanolamine; DSPG, 1,2-distearoyl-sn-glycero-3-phosphatidylglycerol; EPG, 1,2-diacyl-sn-glycero-3-phospho-[1-rac-glycerol]; DPPG, 1,2-dipalmitoyl-sn-glycero-3-phosphorylglycerol; DSPG, 1,2-distearoyl-sn-glycero-3-phospho-(1′-rac-glycerol); DEPC, 1,2-dierucoyl-sn-glycero-3-phosphocholine.

Durg Product Name	Active Ingredient	Formulation	Indication
Doxil Caelyx	Doxorubicin	HSPC, Cholesterol, PEG 2000-DSPE	Ovarian cancer and Kaposi’s sarcoma (KS)
Ambisome	Amphotericin B	HSPC, DSPG, Cholesterol	Fungal infection
Marqibo	Vincristine	ESM, Cholesterol	Non-Hodgkin’s lymphoma and acute lymphocytic leukemia
Onivyde	Irinotecan	DSPC, PEG 2000-DSPE, Cholesterol	Colon cancer
Visudyne	Verteporfin	DMPC, EPG	Choroidal neovascularization
Arikyne	Acamicin sulfate	DPPC, Cholesterol	Bacterial infection
Depocyt	Cytarabine	DOPC, DPPG, Cholesterol, Triolein	Neoplastic meningitis
Depodur	Morphium	DOPC, DPPG, Choles-terol, Triolein	Pain management
Vyxeos	Daunorubicin and Cytarabine	DSPC, DSPG, Cholesterol	Acute myelocytic leukemia
Exparel	Bupivacaine	DEPC, DPPG, Cholesterol, Tricaprylin	Pain management
Lipusu	Paclitaxel	Lecithin, Cholesterol, Threonine, Glucose	Ovarian cancer

In the context of targeted drug delivery utilizing phospholipid vesicles, the incorporation of rigid components such as cholesterol and polymers is essential for enhancing the stability of the drug carrier [101]. This modification serves to prevent drug leakage within the circulatory system, as well as in normal tissues or cells. Therefore, it facilitates controlled drug release at the intended site, extends the circulation time of the drug within the body, and ultimately improves therapeutic efficacy. For instance, Fang et al. [102] demonstrated that embedding rigid polymer nanobowls within the aqueous core of phospholipid vesicles significantly enhanced the stability of doxorubicin-loaded phospholipid vesicles, thereby minimizing drug leakage during blood circulation and augmenting the anti-tumor effectiveness. Similarly, Kohane et al. [103] synthesized aromatic phospholipid vesicles through the covalent attachment of hemolysin lecithin to aromatic fatty acids. This chemical modification resulted in an increase of 19–60% in the drug loading capacity for the hydrophilic local anesthetic tetrodotoxin while also markedly reducing the drug release rate by 30–60% and associated toxicity, thus prolonging the duration of local anesthesia. Furthermore, the use of methyl-branched phospholipids has been shown to promote tighter bilayer packing, which in turn decreases the permeability of the phospholipid vesicles. Kohane et al. [104] found that methyl-branched phospholipid vesicles coated with tetrodotoxin could extend the duration of local anesthesia in vivo and enhance the sustained drug delivery capabilities of the phospholipid vesicle system.

To further advance the clinical applicability of phospholipid vesicles, surface functionalization has been employed to create external stimulus-responsive phospholipid vesicles, which can respond to various stimuli such as light, temperature, ultrasound, or pH [105,106,107,108,109]. These phospholipid vesicle carriers can be activated under specific internal and external stimuli at the target site, thereby improving their permeability and enabling controlled drug release for precision treatment. Li et al. [110] developed targeted heat-sensitive phospholipid vesicles for drug delivery by covalently linking an aptamer that can target tumor cells to a phospholipid molecule modified with polyethylene glycol, in conjunction with a heat-sensitive phospholipid. The experimental results indicated that the hydrophilic polymer polyethylene glycol enhanced the stability of the phospholipid vesicles and reduced the propensity for drug leakage during circulation. The aptamer modification of phospholipid vesicles further increased the active targeting and internalization capabilities of the drug delivery system. Additionally, the temperature-responsive nature of the heat-sensitive phospholipid improved the water solubility of the hydrophobic drug quercetin, enabling precise temperature-controlled release of the drug within tumor cells.

### 3.5. Biological Detection

**Figure 9:**
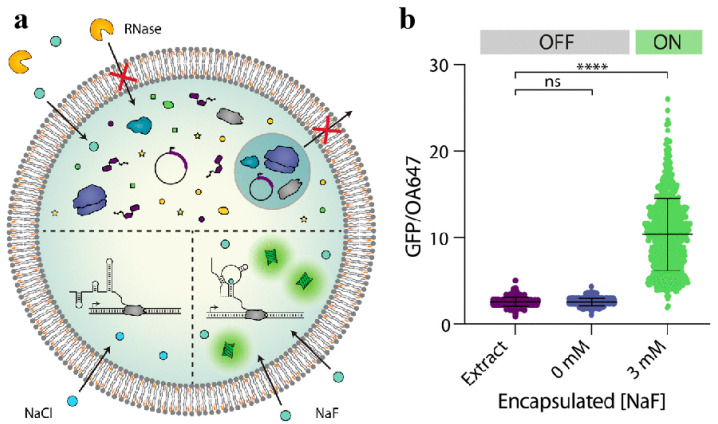
(**a**) Schematic diagram of the specific and sensitive fluoride riboswitch-based biosensor. (**b**) The effect of encapsulated fluoride ion on the riboswitch. Reproduced with permission from [111]. Copyright 2023, Boyd, M.A. et al.

Phospholipid vesicles have also attracted significant interest in the fields of biosensing and disease diagnosis, primarily due to their robust encapsulation capabilities and the potential for surface modification with various active groups. Kamat et al. [111] developed large phospholipid vesicles composed of a mixture of cholesterol and 1-palmitoyl-2-oleoyl-sn-glycero-3-phosphocholine that encapsulated a GFP reporter gene and *Escherichia coli* cell lysate through an emulsion phase transfer technique. This led to the creation of a specific and sensitive fluoride riboswitch-based biosensor (Figure 9a). The encapsulation process did not impede gene expression or fluoride detection. When the riboswitch was co-encapsulated with 3 mM sodium fluoride within the GUVs, GFP expression was observed, and the riboswitch was activated in the “ON” state. Conversely, when only fluoride or nucleic acid was encapsulated, GFP expression remained minimal, and the riboswitch was in the “OFF” state (Figure 9b). The phospholipid membrane exhibits selective permeability to fluoride ions, enabling the riboswitches contained within the vesicles to monitor the fluoride ion concentration in the external environment. Furthermore, the phospholipid membrane serves to shield the internal gene expression from potential interference by external ribose-degrading enzymes (Figure 9a). In contrast to the protective properties of phospholipid membranes, Ren et al. [112] fabricated 100 nm phospholipid vesicles by 1,2-dioleoyl-sn-glycero-3-phosphocholine using an extrusion method and incorporated silica nanoparticles loaded with fluorescein isothiocyanate (FITC) and 1,1′-dioctadecyl-3,3,3′,3′-tetramethylindocarbocyanine perchlorate (Dil) into these vesicles. Upon excitation at 488 nm, two distinct fluorescence peaks at 522 nm (FITC) and 568 nm (Dil) were simultaneously detectable. The fluorescence signal of FITC diminished while that of Dil increased, attributed to the resonance energy transfer phenomenon. Phospholipase A2 (PLA2) can specifically disrupt the phospholipid bilayer, resulting in an increased distance between the two fluorochromes that exceeds the critical distance for fluorescence resonance energy transfer (FRET). This disruption leads to an increase in the fluorescence intensity of FITC, acting as the energy donor, and a decrease in the fluorescence intensity of Dil, serving as the receptor in the FRET system. Consequently, the detection of PLA2 can be achieved with high sensitivity and specificity by monitoring the fluorescence signals at 522 and 568 nm.

**Figure 10:**
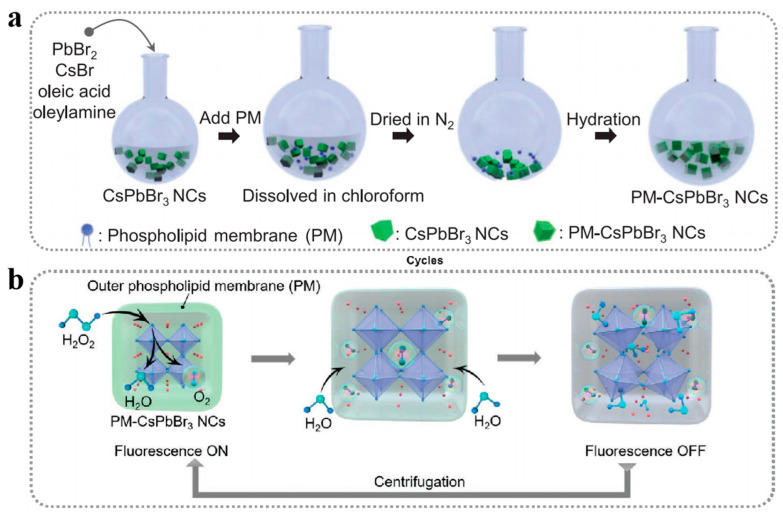
(**a**) Schematic representation of the preparation of phospholipid vesicles that encapsulated hydrophobic CsPbBr_3_ NCs. (**b**) Schematic diagram of the reversible transformation process of phospholipid membrane-coated CsPbBr_3_ NCs before and after H_2_O_2_ treatment. Reproduced with permission from [114]. Copyright 2021, Wiley-VCH GmbH.

Metal ion-based nanomaterials exhibit distinctive catalytic activities that mimic biological enzymes, leading to diverse applications in biosensing, disease treatment, and pollutant remediation [113]. Li et al. [114] synthesized highly biocompatible phospholipid membrane-encapsulated hydrophobic perovskite nanocrystals (CsPbBr_3_ NCs) utilizing a thin film hydration technique (Figure 10a). The experimental results indicated that the phospholipid coating creates a robust protective layer around CsPbBr_3_ NCs, thereby significantly enhancing their stability within biological environments. Upon the gradual addition of hydrogen peroxide (H_2_O_2_), a notable decrease in the fluorescence intensity of CsPbBr_3_ NCs embedded in the phospholipid film was observed, leading to fluorescence quenching and the subsequent decomposition of the perovskite crystals. However, upon the removal of excess H_2_O_2_, the elements that had dissolved within the phospholipid membrane can recrystallize and reattach to the surface of CsPbBr_3_ NCs, resulting in the restoration of fluorescence (Figure 10b). Given that glucose can be oxidized by glucose oxidase to generate H_2_O_2_, the authors leveraged the fluorescence variations exhibited by the phospholipid-encapsulated perovskite nanozymes to quantify the glucose concentrations in blood, thereby highlighting the significant potential of this nanozyme in the domain of bioanalytical applications.

Extracellular vesicles (EVs) are composed of a phospholipid bilayer and are released into various bodily fluids, including serum, plasma, urine, and saliva, by both normal and pathological cells [115]. Due to their capacity to carry specific molecular constituents such as nucleic acids, proteins, and lipids derived from their parent cells [116], EVs are frequently utilized as biomarkers for various diseases, as they can provide an accurate representation of the physiological state of the cell from which they originated [117,118]. In a study from Liu et al. [119], choline phosphate-grafted platinum nanozymes were employed for the immunoassay of urinary extracellular vesicles released by bladder cancer cells. The non-specific interaction between the choline phosphate moiety on the platinum nanozymes and phosphatidylcholine present on the membrane of the extracellular vesicles facilitates the rapid adsorption of the nanozymes onto the vesicle surface. This interaction enhances the signal strength, thereby enabling a precise quantification of the urinary extracellular vesicles secreted by bladder cancer cells.

The integration of diagnostic and therapeutic approaches, which synergistically combines disease diagnosis or monitoring with treatment, presents significant advantages over a single method of disease management. The advancement of a comprehensive strategy that merges a precision drug delivery nanoplatform with real-time high-resolution imaging techniques is crucial for achieving the integration of diagnosis and treatment in cancer and other diseases [120,121].

Huang et al. [122] have developed an intelligent all-in-one therapeutic nanoprobe characterized by glutathione (GSH) sensitivity. This nanoprobe facilitates the precise delivery of the chemotherapy drug temozolomide with micron resolution through fluorescence imaging, enabling localized chemotherapy and the orthotopic tracking of highly immunosuppressive regulatory T lymphocytes. Phospholipid vesicles modified by the polymer PEG effectively prolong the circulation time of nanoprobes in the bloodstream, enhance their accumulation within the tumor microenvironment, and allow for the GSH-sensitive release of temozolomide, thereby promoting precise localized chemotherapy through improved permeability and retention effects. Additionally, the fluorescent dye cyanine7 encapsulated within the phospholipid vesicles generates a robust photoacoustic fluorescence signal, which enables the real-time tracking of the release and accumulation of temozolomide in the tumor microenvironment and facilitates the monitoring of tumor growth. Furthermore, CD25-targeting antibodies incorporated within the vesicles can identify the highly expressed CD25 on T lymphocytes, allowing for the targeting and visualization of changes in T lymphocytes to evaluate immune responses within the tumor microenvironment, thus serving as a tool for the prognostic monitoring of cancer. Yang et al. [123] have developed a novel nanoplatform for phototherapy that is specifically targeted to lysosomes and responsive to pH changes, aimed at facilitating near-infrared II (NIR-II) fluorescence imaging-guided combination therapy involving photothermal therapy (PTT) and photodynamic therapy (PDT) for nasopharyngeal carcinoma. The authors synthesized the NIR-II photothermal agent IRFEM, which incorporates four morpholino groups, demonstrating precise targeting capabilities to lysosomes in nasopharyngeal carcinoma cells. Polymer PEG-modified phospholipid vesicles enhance the permeability and retention of the nanoprobes within the cancer cells, leading to their accumulation in the acidic environment of lysosomes. Upon light irradiation, these vesicles facilitate the release of IRFEM, resulting in the generation of significant amounts of heat and reactive oxygen species. This process engenders specific photophysical interactions whereby PTT and PDT operate synergistically, ultimately inducing cell death through lysosomal mechanisms.

Furthermore, the incorporation of phospholipid vesicles and their surface modifiability allows for the precise delivery of chemotherapeutic agents while simultaneously enabling the real-time monitoring of the tumor microenvironment. This innovative approach represents a promising advancement in the diagnosis and treatment of cancer.

## 4. Summary and Prospect

Leveraging the surface modifiability and amphiphilic characteristics of phospholipids, researchers can engineer functional phospholipid vesicles that exhibit responsiveness to temperature, pH, and light stimuli through the surface modification of small molecules, specific peptides, and polymers. Currently, phospholipid vesicles represent a promising modality for the delivery of therapeutic agents, including drugs and genes. In the contexts of drug administration, gene therapy and biological detection, phospholipid vesicles modified with polymers or proteins enhance the stability of drug carriers, safeguard therapeutic agents from degradation by hydrolase enzymes within lysosomes, facilitate the internalization of drugs and genes, prolong their circulation time, and improve their targeted distribution within the body. Furthermore, these vesicles enable controlled release mechanisms and can synergize with photothermal, photodynamic, and other therapeutic strategies to effectively diagnose and treat cancer.

Despite the notable advancements in biomedical research pertaining to drug delivery, gene therapy, biological detection, and cell mimics, the practical clinical application of phospholipid vesicles for the delivery of drugs, genes, and vaccines remains fraught with challenges. First, phospholipid vesicles are susceptible to hydrolysis by phospholipase in biological systems, necessitating the development of more stable phospholipids to enhance the physical and chemical stability of these vesicles as drug carriers. Second, extracellular barriers, such as the intricate reticuloendothelial system, along with intracellular barriers such as lysosomes, impede the diffusion and biological distribution of phospholipid vesicles that are loaded with therapeutic agents, making it difficult to achieve targeted delivery. By modifying the composition of phospholipids within these vesicles and enhancing the surface modification with proteins, peptides, and polymers, it is possible to improve the targeting and controllability of phospholipid vesicles, thereby facilitating the effective delivery of drugs and gene vectors, thus achieving desired therapeutic outcomes. As research progresses, it is anticipated that the challenges outlined in this discussion will be addressed, ultimately realizing the practical clinical application of phospholipid vesicles in the treatment and diagnosis of various diseases.

## Figures and Tables

**Table 1:** Phase transition temperatures of common phospholipids.

Phospholipid	*T*c (°C)	References
1,2-dilauroyl-sn-glycero-3-phosphocholine (DLPC)	−2	[56]
1,2-dimyristoyl-sn-glycero-3-phosphocholine (DMPC)	23	[54,57,58,59]
1,2-dipalmitoyl-sn-glycero-3-phosphocholine (DPPC)	41	[54,55,59,60]
1,2-distearoyl-sn-glycero-3-phosphocholine (DSPC)	55	[58,59]
1,2-dioleoyl-sn-glycero-3-phosphocholine (DOPC)	−21	[54]
1-palmitoyl-2-oleoyl-sn-glycero-3-phosphocholine (POPC)	4	[54]
1,2-dimyristoyl-sn-glycero-3-phosphoethanolamine (DMPE)	50	[61]
1,2-dipalmitoyl-sn-glycero-3- phosphoethanolamine (DPPE)	66	[62]
1,2-dimyristoyl-sn-glycero-3- phosphorylglycerol (DMPG)	23	[60]
1,2-dioleoyl-sn-glycero-3- phosphorylglycerol (DOPG)	−18	[60]
1,2-dimyristoyl-sn-glycero-3-phospho-L-serine (DMPS)	35	[63]
1,2-dioleoyl-sn-glycero-3- phospho-L-serine (DOPS)	−11	[64]
egg sphingomyelin (ESM)	38	[34,65]

## Data Availability

Not applicable.
